# Whole genome methylation array analysis reveals new aspects in Balkan endemic nephropathy etiology

**DOI:** 10.1186/1471-2369-14-225

**Published:** 2013-10-16

**Authors:** Rada Staneva, Blaga Rukova, Savina Hadjidekova, Desislava Nesheva, Olga Antonova, Plamen Dimitrov, Valeri Simeonov, Georgi Stamenov, Rade Cukuranovic, Jovana Cukuranovic, Vladislav Stefanovic, Momir Polenakovic, Ivanka Dimova, Ruslan Hlushchuk, Valentin Djonov, Angel Galabov, Draga Toncheva

**Affiliations:** 1 Department of Medical Genetics, Medical University of Sofia, 1421 2Zdrave str, Sofia, Bulgaria; 2 National Center of Public Health and Analyses, 15 Acad. Ivan Evst. Geshov blvd, 1431 Sofia, Bulgaria; 3 Vratza District Hospital, 66 “Vtori iuni” blvd, Vratza, Bulgaria; 4 Faculty of Medicine, Institute of Nephrology, University of Nis, Univerzitetski trg 2, 18000 Nis, Serbia; 5 Macedonian Academy of Sciences and Arts, Bul. Krste Misirkov, 2, P.O. Box 428, 1000 Skopje, Republic of Macedonia; 6 Institute of Anatomy, Bern University, Baltzerstrasse 2, CH 3012 Bern, Switzerland; 7 Institute of Microbilogy, Bulgarian Academy of Sciences, 26 Georgi Bonchev str, 1113 Sofia, Bulgaria; 8 Nadezhda Center for reproductive help, 3 Blaga Vest str, 1330 Sofia, Bulgaria

**Keywords:** Epigenetics, Whole genome array analysis, Balkan endemic nephropathy

## Abstract

**Background:**

Balkan endemic nephropathy (BEN) represents a chronic progressive interstitial nephritis in striking correlation with uroepithelial tumours of the upper urinary tract. The disease has endemic distribution in the Danube river regions in several Balkan countries.

DNA methylation is a primary epigenetic modification that is involved in major processes such as cancer, genomic imprinting, gene silencing, etc. The significance of CpG island methylation status in normal development, cell differentiation and gene expression is widely recognized, although still stays poorly understood.

**Methods:**

We performed whole genome DNA methylation array analysis on DNA pool samples from peripheral blood from 159 affected individuals and 170 healthy individuals. This technique allowed us to determine the methylation status of 27 627 CpG islands throughout the whole genome in healthy controls and BEN patients. Thus we obtained the methylation profile of BEN patients from Bulgarian and Serbian endemic regions.

**Results:**

Using specifically developed software we compared the methylation profiles of BEN patients and corresponding controls and revealed the differently methylated regions. We then compared the DMRs between all patient-control pairs to determine common changes in the epigenetic profiles.

*SEC61G, IL17RA, HDAC11* proved to be differently methylated throughout all patient-control pairs. The CpG islands of all 3 genes were hypomethylated compared to controls. This suggests that dysregulation of these genes involved in immunological response could be a common mechanism in BEN pathogenesis in both endemic regions and in both genders.

**Conclusion:**

Our data propose a new hypothesis that immunologic dysregulation has a place in BEN etiopathogenesis.

## Background

Balkan endemic nephropathy (BEN) represents a chronic interstitial nephritis confined to certain regions on the Balkan peninsula - spreading in villages in Serbia, Romania, Croatia, Bosnia and Herzegovina and Bulgaria [1,2]. BEN has late and subtle onset, between the 40s and 60s, with an extensive preclinical period, affecting both genders with slight female predominance. The disease shows familial clustering with affected individuals in several subsequent generations [3]. BEN has a slow progression leading often to terminal kidney failure. The disease has a striking correlation with uroepithelial tumours of the upper urinary tract - about 30-40% of the affected BEN individuals develop such a type of tumour [4,5]. The tumours show varying degrees of malignancy and are mostly papillar carcinomas [5]. They are one of the most common causes of death in BEN patients.

The etiology of BEN still remains elusive. Evidence supporting the involvement of environmental factors is inconclusive. Various chemical elements, organic and non-organic compounds, viruses and microorganisms are implicated in BEN development. Statistically significant differences in soil concentration of heavy metals such as Mg, Mo, Cd, Pb As, Se, Ca, Cu are observed between endemic and non-endemic regions [6], although there is no direct link of a toxic effect of any heavy metal to the disease development. Since BEN has similarities in pathomorphological characteristics with Chinese herbal nephropathy, a common etiology (toxic effect of the aristolochic acid) of both disorders was proposed. There is no irrefutable evidence supporting the effect of aristolochic acid on BEN development. Nevertheless there is a link between aristolochic acid and malignancies in BEN patients [7]. The mycotoxin ochratoxin A is supposed to have a synergic effect with other agents in BEN development [8]. Although there are different papers reporting on numerous viruses (Picorna virus, Polyoma virus, Herpes simplex 1 and 2, Adenovirus, Hepatitis B, Cytomegalovirus, Epstein-Barr virus) found in kidney samples of BEN patients [9,10], there is no substantial evidence supporting viral etiopathogenesis of BEN.

Familial clustering of BEN suggests a genetic predisposition to the disease. A multifactorial model with polygenic genetic predisposition explains the disease characteristics [11,12]. Previous studies have implicated genes located in cytoband 3q25 - 3q26, genes coding xenobiotic metabolizing enzymes, tumour-suppressor genes and proto-oncogenes. There is evidence that the share of rapid debrisoquine metabolizers is higher in BEN patients than in healthy controls [13], thus polymorphic variants in *CYP2D6* causing sensitivity to various chemical agents are suspected in BEN pathogenesis. Partial *LCAT* deficiency was also studied in the context of BEN, since *LCAT* deficient individuals show evidence of renal tubular injury [14]. Cytogenetic research showed *in vitro* higher folic acid induced chromosomal fragility and more frequent spontaneous chromosomal aberrations [15,16]. Some of the regions expressing fragility contain oncogenes - 1p36 - *C-SRC*, 3p25 – *RAF1,* 3q27*- FIM3*, 6q23 – *MYB*, 1p13-*NRAS,* 6p11-*KRAS1P*.

It is well known that environmental factors can influence genome function without changing the DNA sequence itself - the concept of epigenetics. The epigenetic characteristics are maintained by specific mechanisms that firstly ensure the epigenetic profile through cell generations and secondly allow the cell to perform specific functions (differentiation) and to adapt according to different stimuli [17]. The major epigenetic modifications include DNA methylation, histone modifications, miRNA interference [18]. The dynamics of epigenetic processes allow the cells to respond reversibly and in a precise way to environmental stimuli, but also preserve cell type specific gene programmes. When alteration of epigenetic pattern occurs, diseases such as cancer could occur through pathological gene expression. Epigenetic changes over time display familial clustering [19]. This could explain the clustering of some common diseases in families and actually the epigenetic pattern could be implicated in transmitting a “predisposition” over generations.

DNA methylation is the most widely explored epigenetic mechanism. In mammals DNA methylation occurs mainly in regions with a high content of CpG sites. The greater part of GpGs in the human genome is methylated. The CpG islands located in gene promoter regions and other gene related DNA sequences represent a significant exception [20]. CpG islands are defined as DNA sequences sizing at least 200 bp; the GC content is more than 50%, and with an observed-to-expected CpG ratio greater than 60% [21]. More than 50% of human genes contain a CpG island in their promoter region [20,22]. DNA methylation in promoter regions is mostly associated with gene silencing and lower gene expression levels. CpG island methylation is involved physiologically in genomic imprinting, X inactivation and cell differentiation. As well as a fundament for physiological regulation, DNA methylation processes can be involved in disease. An aberrant methylation profile is observed in different cancers [20,22].

Balkan Endemic Nephropathy clinical characteristics, its epidemiological spread and elusive etiology led us to search the background to BEN predisposition on a new genetic level. Epigenetic modifications being heritable and adaptable at the same time may prove to make a significant contribution to BEN development and may be the link between the effect of environmental factors and genetic composition in BEN progression. In the present study we aimed to investigate the methylation status across the whole-genome in different patient groups, based on gender and endemic region, in comparison to healthy controls from non-endemic regions. Differentially methylated regions (DMRs) were determined in different patient-control pairs and after compiling the DMRs data from all pairs the commonly presented DMRs were determined to be the most promising methylation alterations in BEN. Here we report hypomethylation of the promoters of genes *HDAC11, IL-17RA, SECG61* to be associated with BEN.

## Methods

Our study was designed as a case–control study and is based on comparing methylation profiles among BEN patients and healthy controls. It was approved by Ethical Committees in Bulgaria and Serbia. We obtained peripheral blood samples from 3 series of patients after informed consent was received from every participant. Clinical assessment was performed according to unified criteria and these were applied to all sample cohorts [23]. From all 159 patients 5 ml blood samples were collected in EDTA- containing vacuette containers. All samples were checked for DNA consistency by 1% gel electrophoresis. Samples of unsatisfactory quality were excluded from our study. Serbian- Ethics committee of University of Nis, School of Medicine, Nis, Serbia Bulgarian-Commission of Medical Ethics at the National Center of Hygiene, Medical ecology and Nutriotion, Sofia, Bulgaria.

The first sample cohort was collected by preliminary clinical screening of 2500 people in the Vratza endemic regions in Bulgaria in 2003 that revealed 96 patients - 21 males and 75 females [24]. All subjects were of Bulgarian ancestry, born and living in the endemic region. Genealogical analysis was performed to exclude kinship between any study subjects. Thus 44 female samples and 15 male samples were selected, based on clinical information and DNA quality.

The second sample cohort was collected by a current survey in 2011 in Bulgarian endemic regions that revealed 51 cases of BEN - 28 female and 23 male BEN patients. All subjects were of Bulgarian ancestry, born and living in the endemic region. DNA was extracted by standard phenol-chloroform extraction procedure and stored at −80°C. Overall we included 72 female samples and 38 male samples of Bulgarian ancestry.

The third sample cohort - 49 cases, were collected from Serbian endemic regions. DNA was extracted by DNA extraction kit and stored at −80°C. 19 female samples and 30 male samples of Serbian ancestry were enrolled in our study.

Control samples were collected from non-endemic regions in Bulgaria and Serbia after exclusion of any with a family history of kidney disease. Controls had no kidney disease, no anamnestic data for other chronic illnesses, hypertensive disease or diabetes and were clinically healthy at the time of blood sampling. They were matched according to age and sex to BEN samples. 75 female control samples and 31 male controls from Bulgaria and 33 female and 31male control samples from Serbia were included. DNA was extracted by standard phenol-chloroform extraction procedure and stored at −80°C. All samples were checked for DNA consistency by 1% gel electrophoresis.

### Pools

For all DNA, samples concentration was measured by spectrophotometric assay on NanoDrop 2000c (Thermo Scientific inc.). For all samples standard A260/280 and A260/230 data was recorded - only samples with A260/280 > 1.8 and A260/230 >1.8 were further processed. All samples were tested for DNA integrity by 1% gel electrophoresis. DNA concentrations were brought to 100 ng/μl. 1 μg DNA from every sample was added to the respective pool. All samples were assigned to 8 different pools (Table 1). 60 μl of pool-DNA was thermally fragmented at 95°C for 40 min. to obtain 200-1000 bp DNA fragments. Optimal fragmentation was tested on 1% gel electrophoresis with 5 μl of the fragmented DNA pool sample. 5.5 μg from each pool were subjected to analysis. A final volume of 250 μl was achieved by adding PBS.

**Table 1:** Pools and clinical data for the patients included

**Pool**	**Samples (Number)**	**Median age of diagnosis ± 1 SD**	**Mean duration of BEN ±1SD**	**Present uroepithelial tumors (%)**
Serbian male patients	30	73,9 ± 7,3	13,5 ± 5,7	2 (6.6)
Serbian male controls	31	69,6 ± 8,0	-	0
Serbian female patients	19	68,1 ± 8,8	12,5 ± 5,5	0
Serbian female controls	33	72,1 ± 9,3	-	0
Bulgarian male patients	38	71,2 ± 5,9	7,26 ± 9,9	12,5
Bulgarian female patients	72	68,6 ± 7,3	6,89 ± 0,71	3,6
Bulgarian male controls	31	56,3 ± 8,5	-	0
Bulgarian female controls	75	48,6 ± 8	-	0

### MeDIP and array analysis

200 μl of the fragmented DNA pool sample were subjected to methylation DNA immunoprecipitation (MeDiP) for extraction of the methylated fraction from whole genome DNA according to Agilent protocol (v1.1, 2010). For immunoprecipitation we used Dynabeads® Pan Mouse IgG (Invitrogentm) and Anti-5-Methylcytidine Monoclonal Antibody (Eurogentec).

The remaining 50 μl of the start-sample were stored at −20°C as a reference DNA sample. Both methylated and reference samples were subjected to further DNA extraction procedure with phenol-chloroform. The DNA yield was assessed by Nanodrop2000c. 1-2 μg was estimated to be a good yield. The methylated DNA sample was labelled with Cy5 (Cyanine5-red channel) and the reference sample was labelled with Cy3 (Cyanine3-green channel) using Agilent Genomic DNA enzymatic labelling kit (Agilent inc.). Concentration and dye incorporation were measured by Nanodrop2000c. Over 5 μg was considered a good yield. Dye incorporation was considered satisfactory if Cy3 incorporation was between 18-25 pmol/μg and Cy5 incorporation 7-20 pmol/μg. Hybridization was performed on Agilent methylation DNA array (1x244k), conditions were according to Agilent protocol (20 RPM for 40 hrs). The washing procedure was performed by Agilent washing solutions. Slides were immediately scanned on Agilent scanner G2505.

Data were extracted by Agilent Feature extraction software (v.11.0.1.1) and raw data analysis was performed by Agilent Genomic Workbench Lite (v6.5.0.18). The probe methylation status was assessed by BATMAN assay (**Ba**yesian **t**ool for **m**ethylation **an**alysis). Bayesian deconvolution strategy takes into account the estimated distribution of DNA fragment lengths, so that it can discover the most likely configurations of methylated and unmethylated CpGs in a sequence that explains the observed MeDIP signals. This allows estimation of absolute methylation levels. Results were generated in Excel table. We developed our own software for data mining. We designed software to scan through preliminary data to assess the methylation status of all 27 000 CpG islands. When over 60% of probes were methylated (according to BATMAN call) a CpG-island was defined as “methylated”, and when over 60% of probes were unmethylated (according to BATMAN call) the CpG-islands were considered “unmethylated” [25]. CpG islands with methylated or unmethylated probes in the range 40-60% were considered “intermediate methylation” and since an unequivocal call of methylation status for these CpG islands was not possible we excluded them from further analysis. Thus we were able to determine the absolute methylation status of all CpG-islands.

## Results

A first analysis was performed by comparing the methylation status of corresponding pools- patients vs. controls: 1. Bulgarian female patients (BG-F-pat)/Bulgarian female healthy controls (BG-F-con); 2. Bulgarian male patients (BG-M-pat)/Bulgarian male healthy controls (BG-M-con); 3. Serbian female patients (SER-F-pat)/Serbian female healthy controls (SER-F-con); 4. Serbian male patients (SER-M-pat)/Serbian male healthy controls (SER-M-con). The aim of this analysis was to define the differentially methylated region between patients and healthy controls. These are presumed to be loci that affect cell function and lead to BEN development.

A second analysis was performed to reveal the common differentially methylated regions (DMRs) between several patient-control pairs grouped by: 1. endemic region and 2. gender: DMRs in BG-F/SER-F; DMRs in BG-M/SER-M; DMRs in BG-F/BG-M and DMRs in SER-M/SER-F. We aimed to discover which has a greater significance to BEN - endemic region or gender.

The third level of analysis was performed by comparing DMRs in all subsets of patient-control pairs in order to reveal the common changes in the methylation profile in all patient-control pairs, so that we can define the most prominent methylation deregulated loci in BEN.

### DMRs in patient-control pairs

**Figure 1:**
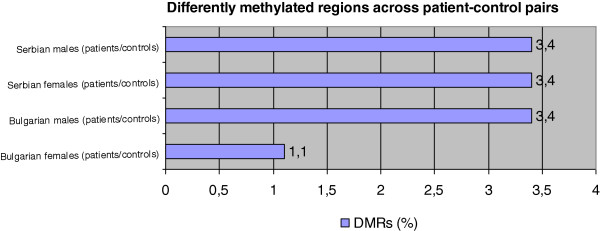
Percentage of DMRs (differently methylated regions) by comparing patients’ methylation profile vs. healthy controls methylation profile.

Our software compared the methylation status of all CpG island and generated a table with only differentially methylated CpG islands (DMRs). Table 2 and Figure 1 show the results of these comparisons. Comprehensive list of all DMRs, their location, related gene and methylation status is provided in supplementary tables. We estimated the number of DMRs in Bulgarian female patients to be 293 (Additional file 1: Table S1), in Bulgarian male patients – 948 (Additional file 2: Table S2), Serbian female patients – 947 (Additional file 3: Table S3), and Serbian male patients – 944 (Additional file 4: Table S4). The percentage of DMRs throughout the DMR is the same in the last 3 pairs – 3.4%. The only significant disparity is the Bulgarian female group (patients/controls) - 1%. This can be attributed to the number of samples in the pool, which is twice as large as the others. This can smooth out the difference in methylation status between patients and controls. As in every pool analysis, in this design also large sample sizes render out small changes in small subsets of patients that can contribute to the disease, but since we are looking for more prominent alterations affecting the greater part of the affected individuals we can classify that as a small drawback.

**Table 2:** Number of DMRs (differently methylated regions) by comparing patients’ methylation profile vs. healthy controls’ methylation profile

**Pool**	**DMRs (Number)**	**DMRs (%)**
Bulgarian females (patients/controls)	293	1.1
Bulgarian males (patients/controls)	948	3.4
Serbian females (patients/controls)	947	3.4
Serbian males (patients/controls)	944	3.4

### Comparing DMRs between different patient-control pairs

We then searched for common DMRs by pairing different group array data. Results are shown in Table 3. Comparing Serbian male/female pairs reveals that they share 313 DMRs from all discovered DMRs (947 in SER-F and 944 in SER-M) - about 33.1% for each group. When comparing Bulgarian males/females this number is less – 65 versus 293 in BG-F (22.2%) and 948 in BG-M (6.9%). One possible explanation is that this can reflect the male to female affected ratio in both populations. In Serbian endemic regions there is not a significant predominance of affected females, while in Bulgarian endemic regions there is a major predominance of affected female BEN patients-2.5:1 [24].

**Table 3:** Common DMRs in different patient-control pairs, based on endemic region and gender

**Subsets of patient-control pairs**	**Number of common DMRs**	**% of common DMRs of all CpG islands**	**% of commonDMRs of defined DMRs in respective group**
Serbian pairs array data (females and males)	313	0.011	33.1/33.1
Bulgarian pairs array data (females and males)	65	0.002	22.2/ 6.9
Both female pairs array data (Bulgarian, Serbian)	45	0.001	15.4/ 4.8
Both male pairs array data ((Bulgarian, Serbian)	167	0.006	17.6/ 17.7

When comparing same gender data BG-F/SER-F (45 DMRs from all DMRs) and BG-M/SER-M (167 DMRs from all DMRs) there is a varying percentage of common DMRs - 15.4% and 4.8% respectively in females and 17.6% and 17.7% (Table 3). In this comparison male patients have a greater percentage of shared common DMRs, so that in general it is difficult draw any conclusion as to whether endemic region or gender is more important to disease pathogenesis.

**Figure 2:**
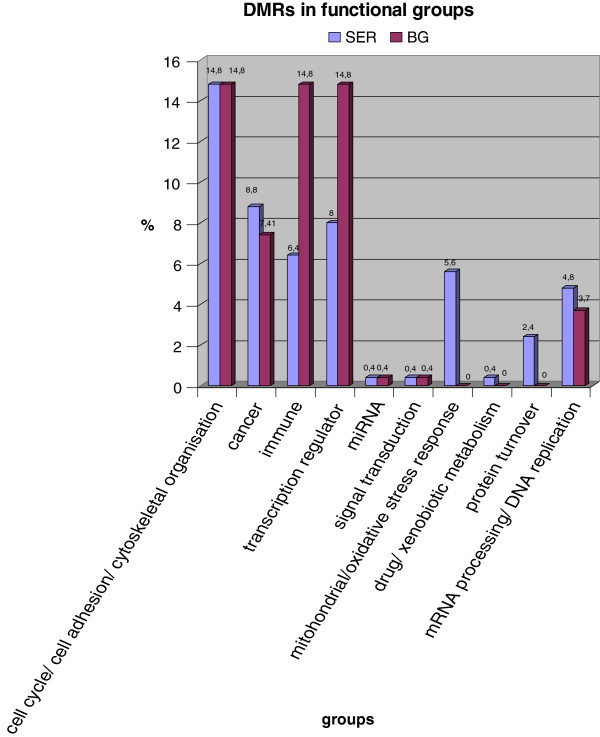
DMRs in groups based on their biological function.

We performed an analysis of the shared DMRs and their associated genes (313 for Serbian arrays and 65 for Bulgarian arrays) according to their function. The functional significance of all genes was ascertained based on several online databases (Genecards - http://www.genecards.org/, OMIM - http://www.omim.org, NCBI - http://www.ncbi.nlm.nih.gov, BioGraph - http://biograph.be). For every gene the primary and secondary biological functions were determined. According to this analysis, all genes were classified in 10 relatively broad groups and their percentage was estimated. Results are presented in Figure 2. In both Bulgarian and Serbian patients major biological processes that appear to be affected are cell adhesion and cytoskeleton organization/regulation of cell cycle - 14.8% in both Bulgarians and Serbians; oncogenesis and metastasis - 8.8% (SER) and 7.41% (BG); immune response – 6.4% (SER) and 14.8% (BG) and transcriptional regulation - 8% (SER) and 14.8% (BG). mRNA processing and DNA replication, signal transduction systems and miRNAs seem to be affected to a lesser extent. It stands out that in Serbian patients the oxidative stress response pathway seems to be involved – 5.6% of DMRs are associated with genes belonging to this pathway. Several of the DMRs – 2.4% (SER) are related to genes in the ubiquitination pathway (protein turnover).

### Comparing DMRs in all four subsets of patients

Since whole genome array analysis generates a vast amount of data, and in order to be sure that our findings are relevant to all patient groups, we compared the DMRs of all four pairs. Only 3 genes has shown to be differently methylated in all pairs - *HDAC11, IL17RA, SEC61G*. All genes are hypomethylated in BEN patients and methylated in healthy controls (Table 4).

**Table 4:** Common DMRs and their related genes in all patient-control pairs

**CpG island (hg19)**	**Start**	**End**	**Cytoband**	**Gene name**	**% hypomethylated probes**	**% hypermethylated probes**
CpG: 60	13521516	13522227	3p25.1	*HDAC11*	60	40
CpG: 158	17565498	17566950	22q11.1	*IL17RA*	60	40
CpG: 56	54826556	54827168	7p11.2	*SEC61G*	60	40

## Discussion and conclusion

This study aims to investigate methylation alterations throughout the whole genome of BEN patients. We report the results of the methylation profiling of BEN patients. Epigenetic methylation status analysis represents a relatively new direction of genetic analysis and genome-wide level methylation analysis is a powerful tool in the search for a genetic background to disease etiology. So far this method has been applied to the study of large scale of disorders varying from different types of cancers [26] to diabetes [27], chronic kidney disease [18,28], rheumatoid arthritis [29] and atherosclerosis [30]. Epigenetics unravels new possible pathogenic mechanisms to common diseases.

The performed methylation analyses on the 1st and 2nd levels reveal a large number of differently methylated loci and prove BEN to be a heterogeneous disease. The separate analysis of the DMRs according to gene function in Bulgarian and Serbian patients revealed several processes to be affected to a similar extent in both groups - cell cycle/cell adhesion and cytoskeleton organization, oncogenesis and metastasis, immune response and transcription regulation. These processes, if deregulated, most likely contribute to a molecular pathogenesis of BEN and a predisposition to uroepithelial tumours. Making a smaller DMRs contribution are miRNA, signal transduction, mRNA processing and DNA replication. It appears that these processes may be deregulated in BEN. Of great interest is the fact that genes involved in innate immune response, inflammation and antiviral immunity seem to be involved in BEN. This is in accordance with the hypothesis of an abnormal immune response to viral and other external stimuli.

In our 3rd level analysis, by comparing the DMRs between all patient-control pairs we discovered 3 genes that are differentially methylated in patients/controls - *HDAC11, IL17RA, SEC61G* These genes represent our most prominent candidate-genes based on the screening we performed.

The CpG island in the promotor area of *HDAC11* is hypomethylated in all four patient groups and hypermethylated in all healthy controls groups. *HDAC11* encodes a class IV histone deacetylase. This is responsible for the deacetylation of lysine residues on the N-terminal part of the core histones (H2A, H2B, H3 and H4). Histone deacetylation tags for epigenetic repression and plays an important role in transcriptional regulation, cell cycle progression and developmental events. Histone deacetylases do not act autonomously but as components of large multiprotein complexes that mediate important transcription regulatory pathways HDACs have a role in cell growth arrest, differentiation and death. The exact biological function is still to be clarified.

*HDAC11* is suggested to have a role not only in normal human tissue processes, but also in the development and progression of human neoplasia [31]. Preliminary studies suggest that the aberrant expression is not due to gene amplification, but rather a result of dysregulation of *HDAC11* gene expression [32], which is consistent with our hypothesis of aberrant hypomethylation leading to excessive activity of HDAC11 and may be involved in the pathogenesis of BEN. HDAC11 is suggested to exist in a protein complex different from the known co-repressor complexes, that participate in other processes apart from strictly modulating chromatin composition [31]. Dysregulation of HDAC11 activity could be a cofactor in tumourogenesis. Inhibition of HDAC11 transcripts is proved to stimulate the production of tumour necrosis-α (TNF-α) and IL-17 in the supernatants of HL ( Hodgkin lymphoma) cells, suggesting invlovement of HDAC11 in immune response modulation. Furthermore, considering the high incidence of urothelial malignancies, we could speculate that HDAC11 deregulation can contribute to a disproportionate immune response and immunological tolerance to malignant cells in BEN patients.

The CpG island in the promotor area of the gene *IL17RA* is hypomethylated in all four patient groups and hypermethylated in all healthy controls groups. It codes a receptor for Il-17 family. The effect of IL-17 family cytokines is mediated by members of the IL-17 receptor family, IL-17 R/IL-17 RA, IL-17 B R/IL-17 RB, IL-17 RC, IL-17 RD, and IL-17 RE. Activation of these receptors triggers intracellular pathways that induce the production of pro-inflammatory cytokines. IL-17A, IL-17 F, and IL-17A/F are produced primarily by activated T cells and signal through an oligomerized receptor complex consisting of IL-17 RA and IL-17 RC. Ligand binding to this complex leads to recruitment of the intracellular adaptor proteins and activation of the transcription factors, NF kappa B, AP-1, and C/EBP. Overexpression of *IL-17RA* can lead to overstimulation of the immune response towards certain peptides by the magnified effect of IL-17A, IL-17E, IL-17C and could facilitate an abnormal inflammation response leading to nephritis. IL-17RA dysregualtion is implicated in major immune-mediated inflammatory disease such as Chron’s disease [33] and psoriatic arthritis [34].

The CpG island in the promotor area of the gene *SEC61G* is hypomethylated in all four patient groups and hypermethylated in all healthy control groups.

The Sec61 complex is the main component of the protein translocation apparatus of the endoplasmic reticulum (ER) membrane [35]. Oligomers of the Sec61 complex form a transmembrane channel where proteins are translocated across and integrated into the ER membrane. SEC61G encodes the gamma-subunit of the complex. Little is known about the role of SEC61 complex in health and disease. According to MOPED data [36] SEC61 complex is expressed in T-lymphocytes and HEK-293 cell line. The fact that *SEC61G* is overexpressed in T-lymphocytes is also in line with our hypothesis of immunological involvement in BEN. *SEC61G* is strongly expressed in HEK293 cell line and bearing in mind that HEK-293 is an embryonic kidney cell line, this may imply that SEC61 is an important factor for early stage kidney cell growth and development. The exact function of this pathway in the adult kidney still remains to be investigated.

In conclusion, our results suggest that methylation alterations in genes related to dysregulation of immune response can contribute to BEN development. It is worth further investigation of their expression levels in kidney samples from BEN patients in order to verify the pathogenic consequences of their hypomethylated status.

## Abbreviations

BEN: Balkan endemic neohropathy; SEC61G: Protein translocase complex, SecE/Sec61-gamma subunit; IL17RA: 17 receptor, alpha subunit; IL: Interleukin; HDAC11: Histone deacetylase 11; LCAT: Lecithin-cholesterol acyltransferase; CYP2D6: Cytochrome P450, family 2, subfamily D, polypeptide 6; C-SRC: Cytoplasmic tyrosine kinase; RAF1: Murine leukaemia viral oncogene homolog 1; FIM3: Friend-murine leukaemia virus integration site 3 homolog; MYB: V-myb myeloblastosis viral oncogene homolog; NRAS: Neuroblastoma RAS viral (v-ras) oncogene homolog; KRAS1P: V-Ki-ras2 Kirsten rat sarcoma viral oncogene homolog pseudogene 1; miRNA: MicroRNA; DMR: Differentially methylated regions; MeDIP: Methylated DNA immunoprecipitation; IgG: Immunoglobulin, class G; BATMAN: Bayesian tool for methylation analysis; Cy3: Cyanine 3; Cy5: Cyanine 5; RPM: Revolutions per minute; BG-F-pat: Bulgarian female patients; BG-F-con: Bulgarian female healthy controls(), 2; BG-M-pat: Bulgarian male patients; BG-M-con: Bulgarian male healthy controls; SER-F-pat: Serbian female patients; SER-F-con: Serbian female healthy controls; SER-M-pat: Serbian male patients; SER-M-con: Serbian male healthy controls; BG-F: Bulgarian females; BG-M: Bulgarian males; SER-F: Serbian females; SER-M: Serbian males; MOPED: Model organism protein expression database; HL: Hodgkin lymphoma; Bp: Base pairs; PBS: Phosphate buffered saline.

## Competing interests

The authors declare that they have no competing interests.

## Authors’ contributions

OA, PD, VS, RC, JC, VS, and MP: sample collection. VS, RC, JC, VS, MP: clinical assessment. DN, OA: Sample preparation. RS, BR, SH: MeDIP processing. RS, BR: methylation analysis. RS, BR: data analysis processing. ID, VD, AG, DT: study design help. RH, VD, AG, DT: conceived of the study, coordination. All authors read and approved the final manuscript.

## Pre-publication history

The pre-publication history for this paper can be accessed here:

http://www.biomedcentral.com/1471-2369/14/225/prepub

## Supplementary Material

Additional file 1: Table S1 Comprehensive list of all differently methylated regions (DMRs) between Bulgarian female patients and Bulgarian female controls. Click here for file

Additional file 2: Table S2 Comprehensive list of all differently methylated regions (DMRs) between Bulgarian male patients and Bulgarian male controls. Click here for file

Additional file 3: Table S3 Comprehensive list of all differently methylated regions (DMRs) between Serbian female patients and Serbian female controls. Click here for file

Additional file 4: Table S4 Comprehensive list of all differently methylated regions (DMRs) between Serbian male patients and Serbian male controls. Click here for file
